# Impacts of “supermoon” events on the physiology of a wild bird

**DOI:** 10.1002/ece3.5311

**Published:** 2019-06-25

**Authors:** Steven J. Portugal, Craig R. White, Peter B. Frappell, Jonathan A. Green, Patrick J. Butler

**Affiliations:** ^1^ School of Biological Sciences, Royal Holloway University of London Egham Surrey UK; ^2^ Centre for Geometric Biology, School of Biological Sciences Monash University Melbourne Victoria Australia; ^3^ Office of the Dean of Graduate Research University of Tasmania Hobart Tasmania Australia; ^4^ School of Environmental Sciences University of Liverpool Liverpool UK; ^5^ School of Biosciences The University of Birmingham Birmingham UK

**Keywords:** circadian, energy expenditure, lunar cycles, supermoon

## Abstract

The position of the Moon in relation to the Earth and the Sun gives rise to several predictable cycles, and natural changes in nighttime light intensity are known to cause alterations to physiological processes and behaviors in many animals. The limited research undertaken to date on the physiological responses of animals to the lunar illumination has exclusively focused on the synodic lunar cycle (full moon to full moon, or moon phase) but the moon's orbit—its distance from the Earth—may also be relevant. Every month, the moon moves from *apogee*, its most distant point from Earth—and then to *perigee,* its closest point to Earth. Here, we studied wild barnacle geese (*Branta leucopsis*) to investigate the influence of multiple interacting lunar cycles on the physiology of diurnally active animals. Our study, which uses biologging technology to continually monitor body temperature and heart rate for an entire annual cycle, asks whether there is evidence for a physiological response to natural cycles in lunar brightness in wild birds, particularly “supermoon” phenomena, where perigee coincides with a full moon. There was a three‐way interaction between lunar phase, lunar distance, and cloud cover as predictors of nighttime mean body temperature, such that body temperature was highest on clear nights when the full moon coincided with perigee moon. Our study is the first to report the physiological responses of wild birds to “supermoon” events; the wild geese responded to the combination of two independent lunar cycles, by significantly increasing their body temperature at night. That wild birds respond to natural fluctuations in nighttime ambient light levels support the documented responses of many species to anthropogenic sources of artificial light, that birds seem unable to override. As most biological systems are arguably organized foremost by light, this suggests that any interactions between lunar cycles and local weather conditions could have significant impacts on the energy budgets of birds.

## 1 INTRODUCTION

In many animals, alterations to physiological processes and behaviors often occur at times which coincide with optimal conditions for key life‐history events such as reproduction, migration, or molt (Raible, Takekata, & Essmar‐Raible, 2017). This co‐occurrence can be observed at multiple scales, including annual, diurnal, tidal, and solar cycles (Cheeseman, Fewster, & Walker, 2017). Among these cycles, the position of the Moon in relation to the Earth and the Sun gives rise to several predictable cycles. The lunar synodic cycle (full moon to full moon, or moon phase) has an average length of 29.5 days and causes changes in the geomagnetic field, gravitational pull, and ambient light levels (Lohman & Willows, 1987). These changes are detectable by a wide range of organisms (McDowell, 1969), and the synodic cycle of the moon therefore has a strong influence on the behavior, foraging efficiency, and energy expenditure of organisms (McDowell, 1969; Navarro‐Castilla & Barja, 2014; Smit, Boyles, Brigham, & McKechnie, 2011). The lunar synodic cycle provides cues that are used by numerous marine organisms, for example, to stimulate and synchronize reproduction (Grant et al., 2009), while changes in nighttime ambient light levels have been shown to affect the behavioral strategies of both predator and prey (Penteriani, et al., 2013). Many nocturnally active animals, for example, alter their behavior with the changing light conditions in connection with the lunar synodic cycle, typically a result of either a change in predation risk or a change in prey availability (Lang, Kalko, Romer, Bockholdt, & Dechmann, 2006).

While the influence of the synodic lunar cycle on nocturnal animals is perhaps intuitive, there is emerging evidence that diurnally active animals can also be influenced by lunar cycles. For diurnal species, full moon events will coincide with the sleep phase of the 24‐hr cycle. Many behaviors, including sleep‐wake cycles, calling, activity, and foraging patterns are controlled by the circadian system (Gwinner, 1996). Most circadian rhythms are endogenously controlled and synchronized by the local light‐dark cycle of the environment (Gwinner, 2003; Tarlow, Hau, Anderson, & Wikelski, 2003). For most diurnal species, there is a characteristic increase in body temperature and heart rate at dawn, both of which remain elevated during the active daytime period, and are then significantly lower at night during the rest period (Brown et al., 2002). These likely reflect a state of reduced energy expenditure at night for diurnal animals. In some species of diurnal endotherms, this circadian rhythm in body temperature can include bouts of nighttime torpor, a state of decreased endogenous heat production and lowered body temperature which is used to further conserve energy (Butler & Woakes, 2001; Calder & Booser, 1973; Cheke, 1971). Bright nights may, therefore, have significant implications for the daily energy expenditure and energy budget of diurnal species if their typical circadian rhythms of rest and activity are interrupted.

**Figure 1:**
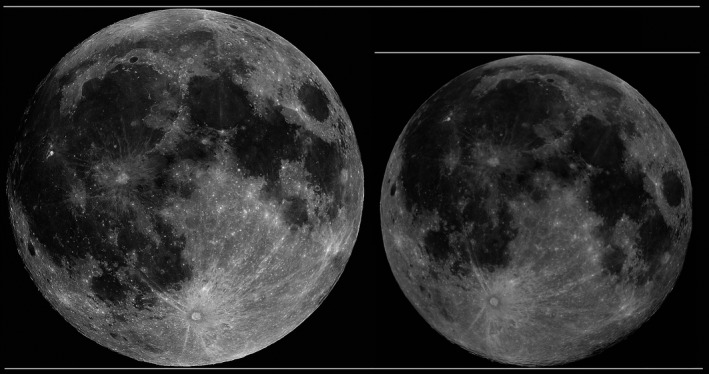
Perigee and Apogee. Calibrated images of perigee (left hand side) and apogee (right hand side) showing the size difference in the sky between the two opposing lunar distances. At time of capture of the images, the distance from Earth for the perigee moon (left) was 356,790 km, at an altitude of 68.82° (1.2^c^). For the apogee moon (right), the distance was 406,357 km from Earth, at an altitude of 44.87° (0.78^c^). Photo taken by, and used with permission of, Anthony Ayiomamitis. Image taken in Athens, Greece, on the 30 January 2010 and 25 August 2010, for perigee and apogee, respectively. The white solid lines are for ease of comparison of the two apparent moon sizes

Increases in nighttime ambient light levels can be caused either by, (a) the synodic lunar cycle, with full moons being brighter, or (b) the moon's orbit, the distance of the moon from the Earth. The limited research undertaken to date on the physiological responses of animals to the lunar illumination has exclusively focused on the synodic lunar cycle but the moon's orbit may also be relevant. The moon's distance from Earth varies throughout its monthly path because the moon's orbit is not perfectly circular (Chapront‐Touze and Chapront, 1983). Every month, this eccentric orbit carries the moon to *apogee*, its most distant point from Earth—and then to *perigee,* its closest point approximately two weeks later. During the perigee phase, the moon is ~46,000 km closer to the earth than at apogee (Chapront‐Touze and Chapront, 1983) and it appears significantly larger in the sky (Figure 1). Since this cycle—the lunar distance—operates independently of the synodic lunar cycle, when perigee occurs in conjunction with a new moon approximately three times a year, a “supermoon” is observed (Kyba, Mohar, & Posch, 2017). During a “supermoon” (the perigee syzygy), the light from the moon will be at its brightest—approximately 30% brighter—and has the potential to have a further impact on the circadian physiology of animals. A typical summer full moon, for example, at temperate latitudes provides only about 0.05–0.1 lux, compared a “supermoon” in the tropics directly overhead potentially providing up to 0.36 lux (Kyba et al., 2017). However, this potential influence has never previously been investigated, and has yet to be integrated into the synodic lunar cycle, in order to fully understand how these two cycles interact to influence the physiology of animals. Here, to study the influence of multiple interacting lunar cycles on the physiology of diurnally active animals, we use migratory barnacle geese (*Branta leucopsis*) as an exemplar species. Barnacle geese are visual‐feeding herbivorous terrestrial grazers, exhibiting a strict diurnal behavioral pattern during the winter months when in Scotland of feeding on salt marsh and farmland during the day, and roosting on water at night (Phillips et al., 2003). Our study, which uses biologging technology to study body temperature and heart rate, asks whether there is evidence for a physiological response to natural cycles in lunar brightness, particularly “supermoon” phenomena, in wild birds.

## 2 MATERIALS AND METHODS

### 2.1 Birds

Eight wild adult barnacle geese (mean body mass, 2.1 ± 0.6 kg) were caught at Ny‐Ålesund research station on the island of Spitsbergen in the Svalbard archipelago (788,550 N, 118,560 E) during the flightless period of their annual wing molt in July 1999 (see Portugal, Thorpe, Green, Myatt, & Butler, 2009; Portugal, Butler, Green, & Cassey, 2011 for molt definitions and further details). All captured geese were color‐ringed to aid recapture in 2000. The population that breeds at Ny‐Ålesund winter on the Solway Firth (south‐west Scotland, 54.981253, −3.489971), and migrate along the Norwegian coast during their annual autumn migration (Butler, Woakes, & Bishop, 1998). During the winter period, from October to April, the Svalbard population of barnacle geese occupy the smallest known area for a wild goose population in the world (Phillips et al., 2003), predominantly inhabiting the Wildfowl and Wetlands Trust (WWT) reserve at Caerlaverock. The birds roost approximately half a kilometer offshore within the Solway estuary on salt marsh, and are thus safe from land predators such as red foxes (*Vulpes vulpes*). Thus, although the precise locations of the eight geese were not known, we can be confident that they were feeding and roosting within a known area of ~100 km^2^. The birds then depart Scotland late April, and travel North to Svalbard over a period of 5–6 weeks. During the summer in Svalbard, the geese are exposed to 24‐hr daylight, with light levels never dropping below civil twilight.

### 2.2 Body temperature and heart rate measurements

**Figure 2:**
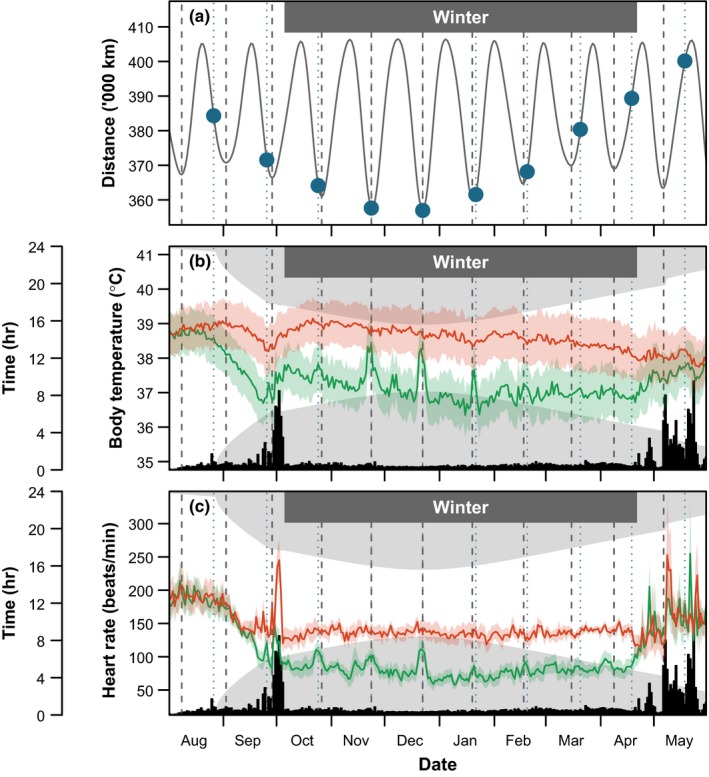
The influence of the synodic lunar cycle and lunar distance on body temperature and heart rate in six barnacle geese. (a) Circannual variation in lunar distance (solid gray line), with the dates of full moons indicated with blue points and the winter study period indicated. Circannual variation in daytime (red) and nighttime (green) body temperature (b) and heart rate (c). Full moons are indicated in all panels with vertical blue dotted lines, and minimum lunar distances are indicated with vertical dashed gray lines. Day length is indicated by shading and circannual variation in activity (hr) is shown as black bars in panels (b) and (c). Activity rates were calculated from heart rate data (see Section 2). The autumn migratory flight from Svalbard to Scotland takes place at the end of September, and the geese were still in Scotland mid‐April. Analyses were conducted on abdominal body temperature and heart rate data from 6 October 1999 to 20 April 2000

Abdominal body temperature (*T*_ab_) and heart rate (*f*_H_) were measured continuously throughout the annual cycle by custom‐made implantable heart rate data loggers (Butler & Woakes, 2001), successfully used with this species on previous occasions (Butler et al., 1998; Portugal, Thorpe, et al., 2009; Portugal, White, Green, & Butler, 2019; Ward, Bishop, Woakes, & Butler, 2002). The loggers (5 cm × 2.5 cm × 0.7 cm) were programmed to record every 5 s, and ran from early August 1999 to mid‐May 2000 (Figure 2). Recordings of *T*_ab_ and *f*_H_ for a minimum of 9 months were obtained from all six birds that were recaptured in the summer of 2000; the remaining two birds were resighted but recapture was not possible. The six geese were failed breeders in 1999 prior to capture; although eggs were laid, they were not successfully hatched.

Methods for logger implantation and removal are described in detail elsewhere (Bevan, Woakes, Butler, & Croxall, 1995; Guillemette et al., 2016). Before use, the loggers were encased in paraffin wax and encapsulated in silicon rubber to provide waterproofing and bio‐compatibility. The temperature sensor of the encapsulated data logger was calibrated by immersing the device in water baths of known temperature. The mass of the internally implanted device (21 g) was comfortably below the 5% of total body mass recommended when deploying internally implanted biologging devices on birds (Portugal & White, 2018; White et al., 2013). Mean daily heart rate of all geese was used to determine daily activity levels (see Portugal, Green, White, Guillemette, & Butler, 2012), in order to identify the autumn migratory flights taking place in September, from Svalbard to Scotland. Nighttime mean *T*_ab_ and *f*_H_ were calculated on an individual basis as the mean between 23:00 and 03:00. Daytime mean *T*_ab_ and *f*_H_ were calculated as means between 09:00 and 15:00.

### 2.3 Meteorological and astronomical data

Minimum and maximum temperatures (°C), precipitation (mm), and cloud cover (%) were acquired as daily means for the winter study period (October 6, 1999 until 20 April 2000) from the British Meteorological Office, obtained from a weather station based at Eskdalemuir (55.267751, −3.175316), a 20 km (beeline) distance from the WWT Caerlaverock reserve. Cloud cover data were cross‐referenced with local newspaper archives, and if a discrepancy of more than 10% in cloud cover was observed between the local archives and the British meteorological office, a mean was taken (*N* = 7). If the discrepancy was <10%, the percentage cloud cover value from the British meteorological office was used. Lunar data (synodic phase and distance from Earth) were calculated using the lunar package in R (Lazaridis, 2014; R Core Team, 2017). We calculated lunar distance as a continuum between, and including, perigee and apogee, scaled to fall between 0 [nearest distance over the observation period] and 1 [furthest difference during the observation period]), and synodic phase (calculated as the absolute value of cosine of the lunar phase angle, in radians, minus π, such that a value of zero corresponds to the full moon and a value of π corresponds to a new moon). Daylength was calculated using the geosphere package in R (Forsythe, Rykiel, Stahl, Wu, & Schoolfield, 1995).

### 2.4 Analysis

Preliminary analyses revealed among‐bird variation in all measures of both *T*_ab_ and *f*_H_, as well as substantial within‐bird temporal autocorrelation in both *T*_ab_ and *f*_H_. Data were therefore analyzed using a linear mixed model framework to account for the among‐individual differences, and to model the temporal autocorrelation explicitly. Linear mixed models were implemented in the nlme package of R (Pinheiro & Bates, 2000; R Core Team, 2017), with variances associated with the random effects estimated by Maximum Likelihood. We modeled both nighttime and daytime *T*_ab_ and *f*_H_ as dependent variables, for a total of four models. All models included lunar distance, synodic phase, cloud cover (%), and the two‐ and three‐way interactions between these as fixed effects, to determine whether these (as determinants of lunar illumination) had a significant effect on *T*_ab_ or *f*_H_. All models also included minimum temperature (°C) and precipitation (mm) as fixed effects, because of the possible influence of these on *T*_ab_ and *f*_H_ (Bryant & Westerterp, 1983; Moreno, 1989; White, Blackburn, Martin, & Butler, 2007; White, Grémillet, Green, Martin, & Butler, 2011). Date was included as a continuous fixed effect in all models to account for potential trends in *T*_ab_ or *f*_H_ through time, and all models also included a random intercept for individual ID, and an order 1 temporal autocorrelation structure for each bird. Fixed effects and interactions were considered significant at *p* < 0.05. Variance inflation factors for the interaction terms in these models were always high (greater than 10 in all cases), indicating that multicollinearity among predictors results in inflated estimates of standard errors and conservative tests of significance (O'Brien, 2007). Conclusions regarding significant interactions are therefore robust, but caution is warranted when interpreting the nonsignificant interactions. So nonsignificant interactions (all data is available in the supplementary material).

## 3 RESULTS

Barnacle geese are exposed to constant daylight during the summer breeding season at Svalbard, exhibit a period of seasonal hyperthermia prior to their autumn migration, and the migration itself is characterized by high heart rates (Butler & Woakes, 2001; Figure 2). Although the geese experienced perigee events during their summer season at Svalbard (Figure 2a), the analysis was restricted to the winter period when the birds were at Caerlaverock (Scotland, UK; Section 2) to exclude the possible effects of long days, migration, and seasonal hypothermia on responses to the lunar cycle. During the 1999–2000 winter season (October 6, 1999 until 20 April 2000), when the barnacle geese were at Caerlaverock, they experienced seven perigee events (Figure 2a). These perigee events varied in distance by up to 10,000 km, with the minimum perigee distance between the Moon and Earth occurring during November, December, and January, and further perigee events occurring during October, February, March, and April. Full moons can occur at any point along the Moon's elliptical path, but are referred to as a “supermoon” when the new moon is an exact match (i.e., the same day) in time with perigee. This occurred during all three closer approaches in November, December, and January. The perigees in October and February would be defined as a “near match” (2–3 days either side; Figure 2a; Kyba et al., 2017).

**Figure 3:**
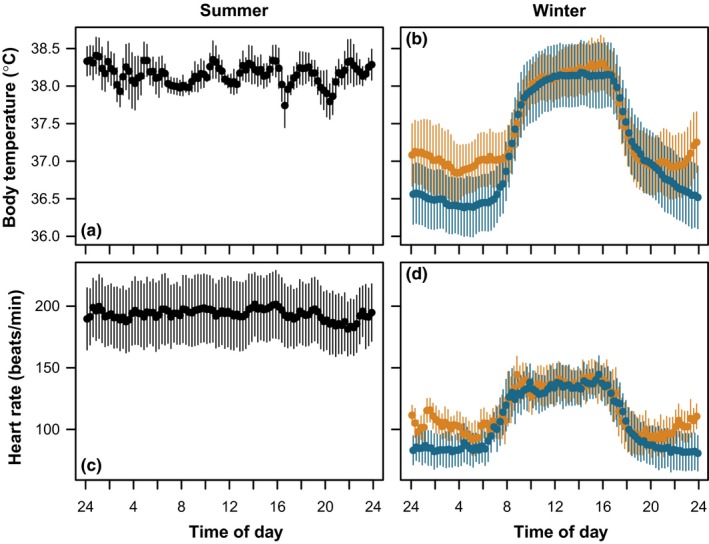
Diel variation in body temperature and heart rate in six barnacle geese. Data for summer are shown in panels (a) and (c), data for winter are shown in panels (b) and (d) and are divided into days with “supermoons” when full moons coincided with perigee (orange) and days in which new moons coincided with apogee (“micromoons”) (blue)

The geese exhibited a pronounced circadian rhythm in heart rate (*f*_H_) and abdominal body temperature (*T*_ab_), with both increasing at dawn and decreasing at dusk during the winter months (Figure 3). There was no obvious rhythm in either physiological parameter during the constant daylight of Svalbard (Figure 3), but Lombe‐Scargle periodograms (Ruf, 1999) revealed rhythms with a periodicity of ~24 hr in two of five birds for *f*_H_, and four of five birds for *T*_ab_.

**Figure 4:**
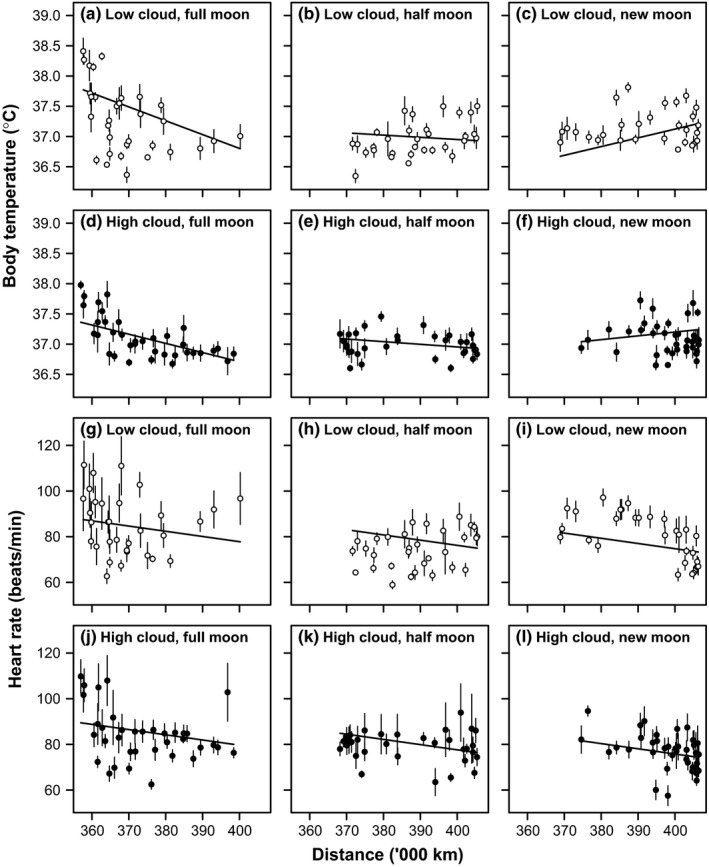
The relationship between nighttime mean (a–f) body temperature and lunar distance, and (g–l) heart rate and lunar distance in six barnacle geese. To explore the significant three‐way interaction between the synodic lunar cycle, lunar distance, and cloud cover for body temperature (Table 1), the data are subdivided by cloud cover (<50%: a–c, g–i, unfilled symbols; ≥50%: d–f, j–l, filled symbols) and by synodic lunar cycle (Full moons: a, d, g, j, waxing or waning moons: b, e, h, k, new moons: c, f, i, l). For presentation, data points are adjusted for consistent inter‐individual differences and shown and mean ± *SE*, and moon phases were separated as follows: for full moons (a, d, g, j), phase angle in radians is within π/3 of full moon; for new moons (c, f, i, l), phase angle is within π/3 of a new moon; for waxing or waning moons (b, e, h, k), phase angles are intermediate. Solid lines are derived from parameter estimates for the full model (Table 1) for heart rate, calculated for a cloud cover of 0% for low cloud (a–c) and 100% for high cloud (d–f) and for a full moon (a, d), half‐moon (b, e), or new moon (c, f). For body temperature, parameter estimates are calculated for a reduced model excluding two‐ and three‐way interactions that are nonsignificant in Table 2, calculated for a cloud cover of 0% for low cloud (g–i) and 100% for high cloud (j–l) and for a full moon (g, j), half‐moon (h, k), or new moon (i, l)

There was a three‐way interaction between lunar phase, lunar distance, and cloud cover as predictors of nighttime mean *T*_ab_ (Table 1). The result of this was that the negative effect of distance on nighttime mean *T*_ab_ was strongest during full moons and clear nights, when the full moon coincided with perigee moon (Figure 4a–f), leading to elevations in nighttime mean *T*_ab_ (Figure 3b). There was a negative relationship between nighttime mean *T*_ab_ and precipitation, and nighttime mean *T*_ab_ declined significantly throughout the study period (Table 1). There was no effect of cloud cover, lunar distance, or lunar phase on daytime mean *T*_ab_, which exhibited a negative relationship with precipitation and declined throughout the study period (Table 2).

**Table 1:** The effects of cloud cover (Cloud), lunar distance (Distance), lunar phase (Phase), minimum ambient temperature (Temperature, °C), precipitation (Precipitation, mm), and date (Date) on nighttime mean body temperature (°C) in six barnacle geese

	Value	*SE*	*t*‐value	*p*‐value
Intercept	**66.6**	**5.29**	**12.6**	**<0.0001**
Cloud	**−0.0043**	**0.0010**	**−4.15**	**<0.0001**
Distance	**−1.16**	**0.20**	**−5.96**	**<0.0001**
Phase	**−0.44**	**0.07**	**−5.97**	**<0.0001**
Temperature	−0.0013	0.0028	−0.46	0.65
Precipitation	**−0.0026**	**0.0011**	**−2.27**	**0.02**
Date	**−0.0026**	**0.0005**	**−5.46**	**<0.0001**
Cloud × Distance	0.0041	0.0023	1.73	0.08
Cloud × Phase	**0.031**	**0.001**	**3.52**	**0.0004**
Distance × Phase	**0.62**	**0.11**	**5.76**	**<0.0001**
Cloud × Distance × Phase	**−0.0028**	**0.0012**	**−2.26**	**0.02**

Notes

Autocorrelation parameter = 0.68. Variance estimates for the random effects: intercept: 2.25, Residual: 0.19. Degrees of freedom for all parameters = 1,247. Significant effects are highlighted in bold.

**Table 2:** The effects of cloud cover (Cloud), lunar distance (Distance), lunar phase (Phase), minimum ambient temperature (Temperature, °C), precipitation (Precipitation, mm), and date (Date) on daytime mean body temperature (°C) in six barnacle geese

	Value	*SE*	*t*‐value	*p*‐value
Intercept	**79.8**	**4.81**	**16.6**	**<0.0001**
Cloud	−0.00050	0.00052	−0.97	0.33
Distance	0.046	0.11	0.41	0.69
Phase	−0.005	0.044	−0.11	0.91
Temperature	−0.0016	0.0014	−1.10	0.27
Precipitation	**−0.0013**	**0.0006**	**−2.21**	**0.03**
Date	**−0.0038**	**0.0004**	**−8.66**	**<0.0001**
Cloud × Distance	0.0006	0.0012	0.56	0.57
Cloud × Phase	0.0052	0.00043	1.20	0.23
Distance × Phase	0.018	0.06	0.30	0.76
Cloud × Distance × Phase	−0.00057	0.00062	−0.92	0.36

Notes

Autocorrelation parameter = 0.81. Variance estimates for the random effects: intercept: 2.33, Residual: 0.08. Degrees of freedom for all parameters = 1,247. Significant effects are highlighted in bold.

Although *f*_H_ appeared to vary with the lunar phase of the synodic cycle and the distance of the Moon from Earth in a similar pattern to *T*_ab_ (Figure 2c), there were no significant two‐ or three‐way interactions between cloud cover, lunar distance, or lunar phase on nighttime mean *f*_H_ (Table 3). There was, however, a significant main effect of distance, such that *f*_H_ decreased with lunar distance irrespective of cloud cover or lunar phase, although the relationship was weak (Figure 4g–l). There was also a significant positive relationship between nighttime mean *f*_H_ and mean ambient temperature (Table 3). There was no effect of cloud cover, lunar distance, or lunar phase on daytime mean *f*_H_, which also exhibited a positive relationship with precipitation (Table 4).

**Table 3:** The effects of cloud cover (Cloud), lunar distance (Distance), lunar phase (Phase), minimum ambient temperature (Temperature, °C), precipitation (Precipitation, mm), and date (Date) on nighttime mean heart rate (beats/min) in six barnacle geese

	Value	*SE*	*t*‐value	*p*‐value
Intercept	140.6	212.2	0.66	0.51
Cloud	0.021	0.037	0.57	0.57
Distance	**−17.7**	**7.2**	**−2.44**	**0.01**
Phase	−4.66	2.74	−1.70	0.09
Temperature	**0.24**	**0.10**	**2.34**	**0.02**
Precipitation	−0.07	0.04	−1.71	0.09
Date	−0.004	0.019	−0.23	0.82
Cloud × Distance	−0.06	0.08	−0.72	0.47
Cloud × Phase	−0.01	0.03	−0.34	0.74
Distance × Phase	4.98	3.96	1.26	0.20
Cloud × Distance × Phase	0.038	0.044	0.87	0.39

Notes

Autocorrelation parameter = 0.68. Variance estimates for the random effects: intercept: 525, Residual: 386. Degrees of freedom for all parameters = 1,234. Significant effects are highlighted in bold.

**Table 4:** The effects of cloud cover (Cloud), lunar distance (Distance), lunar phase (Phase), minimum ambient temperature (Temperature, °C), precipitation (Precipitation, mm), and date (Date) on daytime mean heart rate (beats min^‐1^) in six barnacle geese

	Value	*SE*	*t*‐value	*p*‐value
Intercept	127.7	141.7	0.90	0.37
Cloud	−0.049	0.039	−1.24	0.21
Distance	−5.40	6.57	−0.82	0.41
Phase	0.50	2.44	0.21	0.84
Temperature	**−0.21**	**0.10**	**−1.99**	**0.046**
Precipitation	**0.18**	**0.04**	**4.20**	**<0.0001**
Date	0.001	0.013	0.07	0.94
Cloud × Distance	0.12	0.09	1.38	0.17
Cloud × Phase	0.018	0.033	0.55	0.58
Distance × Phase	0.04	3.63	0.01	0.99
Cloud × Distance × Phase	−0.06	0.05	−1.31	0.19

Notes

Autocorrelation parameter = 0.57. Variance estimates for the random effects: intercept: 525, Residual: 386. Degrees of freedom for all parameters = 1,234. Significant effects are highlighted in bold.

## 4 DISCUSSION

Our study is the first to report the physiological responses of wild birds to “supermoon” events. The wild geese responded to the combination of two independent lunar cycles, by significantly increasing their body temperature (*T*_ab_) during “supermoon” events. Given the role that the daily, lunar, and seasonal cycles of light have played in driving the development of physiological pathways and individual behavior (Gaston, Bennie, Davies, & Hopkins, 2013), it is surprising birds have not evolved a way of ignoring natural celestial events such as “supermoons” and bright light to save increasing *T*_ab_ and *f*_H_. That wild birds respond to natural fluctuations in nighttime ambient light levels support the documented responses of many species to anthropogenic sources of artificial light, that birds seem unable to override (Gaston et al., 2013). As most biological systems are arguably organized foremost by light (Gaston, Visser, & Holker, 2014), this suggests that any interactions between lunar cycles and local weather conditions (e.g., cloud cover) could have significant impacts on the energy budgets of birds.

It is likely that the combination of the moon being full and close to the Earth caused a “masking” effect, whereby the higher ambient light levels acted as a stimulus to cause an immediate overriding of the typical endogenous circadian clocks of the geese (see Kronfeld‐Schor et al., 2012, for a description of masking effects). During a full moon, for example, mRNA levels of cryptochrome (*Cry*)—one of the key circadian clock genes—are significantly elevated compared to those during the rest of the lunar cycle in numerous species of coral (Anthozoans) and fish (e.g., *Siganus guttatus*), providing a potential underlying mechanism for behavioral changes observed in response to the lunar cycle (Fukoshiro et al., 2011). The mechanism behind the changes observed in *T*_ab_ in the geese is likely to be the interruption of normal melatonin rhythms, which causes extensive disruptive effects linking multiple body systems (Goto, Oshima, Tomita, & Ebihara, 1989). Light is a vital component for the regulation of both daily and seasonal processes in birds, and it has been suggested that light during the nighttime may alter the ability of bird's to detect changes in daylength the following day (Longcore & Rich, 2004; Titulaer, Spoelstra, Lange, & Visser, 2012). Being receptive to changes in daylength—photosensitivity—is the primary stimulus (*zeitgeber*) for many key annual events such as migratory departure dates and the onset of molt (Gwinner, 2003).

Melatonin is one of the fundamental hormones involved in the regulation of daily physiological cycles, and is released at night and suppressed by daylight (Gwinner & Hau, 2000). The exogenous application of melatonin directly changes typical circadian rhythms, with Japanese quails (*Cortunix japonica*), for example, altering the standard circadian rhythm in *T*_ab_ following melatonin supplementation (Nakahara, Kawano, Shiota, & Murukami, 2003). This suggests that the proximate mechanism behind increases in *T*_ab_ in the geese during a “supermoon” night is likely due to the suppression of the release of nighttime melatonin (Bentley, 2001; Turek, McMillan, & Menaker, 1976; Ubuka, Bentley, Ukena, Wingfield, & Kazuyoshi Tsutsui, 2005). In experimental settings, the intensity of the nighttime illumination directly determines the degree of the suppression of the melatonin release (Dominoni, Goyman, Helm, & Partecke, 2013). Similarly, Tarlow et al. (2003) demonstrated that full moons significantly inhibit the production of melatonin in Nazca boobies (*Sula granti*), with diel variations in melatonin concentrations vanishing when the moon was full. The increase in brightness of a “supermoon” is likely sufficient to cause substantial changes in the chronobiology of the geese. Future studies into melatonin release and suppression during “supermoon” and perigee events would be fruitful to elucidate the exact mechanisms that cause the rise in *T*_ab_ in the geese. Furthermore, ascertaining whether the birds wake up during “supermoon” and/or perigee events as a result of the higher ambient light levels would give further insight into the potential carry‐over effects of higher light levels at night.

Due to the field‐based nature of this study, it is not possible to determine whether the responses in physiology of the geese are a result of a direct effect of the changes in light levels on the circadian physiology, or via other alternative pathways. Our paper presents novel phenomenological findings that show wild geese physiologically respond to “supermoon” events. Owing to the age of the data (collected in 1999–2000), there are technological limitations that may potentially limit the generality of our findings. Were this study to be repeated now, birds would be deployed with GPS and accelerometry loggers (Gleiss, Wilson, & Shepard, 2011; Halsey, Portugal, Smith, Murn, & Wilson, 2009; Portugal et al., 2014; Taylor, Portugal, & Biro, 2017; Voelkl et al., 2015) in conjunction with the heart rate data loggers, to fully understand where the birds precisely where, and what levels of activity and movement they were exhibiting during the “supermoon” events.

Sleep is a fundamental physiological process (Roth et al., 2010; Vorster & Born, 2014), the deprivation of which could have both proximate and ultimate consequences for the geese. Electroencephalography (EEG) studies on birds during “supermoon” events would be productive, to ascertain whether they are waking up, and to determine the extent of sleep disruption. Similarly, establishing whether “supermoon” events disrupt the previously observed correlations between mean, minimum, and maximum daily heart rates (Portugal et al., 2016) would ascertain the functional significance of sleep interruption at the energetics level. The disruption to the typical circadian *T*_ab_ rhythm in geese occurring in response to natural cycles of ambient light levels may offer insight into how animals are responding to artificial light (De Jong, et al., 2016). Prior studies have demonstrated suppression of rest‐phase torpor in urban birds and mammals (Le Tallex et al., 2015) in response to artificial light, which assumingly must come at a metabolic cost (Guppy & Withers, 1999).

The increased potential risk of being predated upon when the ambient light level is higher means it could be beneficial for animals to wake up during a “supermoon.” Risk of predation during higher light levels can have a significant impact on the behavior of many species. Seabirds such as petrels avoid returning to their breeding colonies on moonlit nights to avoid predation from diurnal predators (Yamamoto et al., 2008), while Galapagos fur seals (*Arctocephalus galapagoensis*) haul out ashore, thought to be in response to the increased risk of predation from visual predators such as sharks (Horning and Trillmich, 1999). Bright nights as a result of a full moon or perigee should favor visually guided predators, compared to predators relying on other senses, such as olfaction or echolocation (Martin, 2010). Some species, such as willets (*Catoptrophorus semipalmatus*), switch from tactile foraging on moonless nights to visually guided foraging on moonlit nights (McNeil & Rompre, 1995), suggesting there is some plasticity in foraging approaches in response to ambient light levels for species which use multiple sensory‐ecological approaches to finding food. The barnacle geese wintering at Caerlaverock forage on agricultural land and roost on water (Phillips et al., 2003). Thus, despite being visually guided foragers, it is very unlikely the geese are waking up to forage on moonlit nights, as there is little to no food available to them in the immediate vicinity of their roosts. Furthermore, unlike *T*_ab_, *f*_H_ does not show such an extreme increase during perigee events as to nearly match daytime *f*_H_ activity levels (Portugal, Green, & Butler, 2007; Portugal et al., 2012, 2019; Figure 2). This small degree of increase in *f*_H_ compared to flight confirms that it is unlikely that the birds are becoming active or moving extensively since even small regular movements are typically accompanied by increases in heart rate (Butler, Green, Boyd, & Speakman, 2004). Despite the geese being generally safe from predators when roosting on or near the water, it is likely that the increased potential risk of being predated upon is one of the drivers for why the geese may be waking up, instigated by the higher ambient light levels masking their typical circadian physiology. Although for this specific population predation at night from ground predators is rare, for other populations and indeed other geese species, it is possible that there may be an elevated risk of being predated upon during supermoon events, particularly by predators relying more on visually guided hunting techniques.

During “supermoon” events, the increases in *T*_ab_ are not matched by significant increases in *f*_H_, and this may be linked to the lack of sustained movement that the geese are undertaking on these nights. Lunar phase has been shown to significantly impact heterothermy in freckled nightjars (*Caprimulgus tristigma*), and thermoregulatory patterns were tightly coupled with the level of nocturnal lunar illumination, rather than ambient temperature (Smit et al., 2011). However, the nightjars became increasingly heterothermic during new moon periods of the lunar cycle, thought to be linked to a reduction in foraging opportunities due to lower light levels, rather than a direct physiological response to the lower light levels themselves. For the barnacle geese in the present study, it is feasible that this occasional increases *T*_ab_ and *f*_H_ have little impact on the energy budgets of healthy adult barnacle geese in good physiological condition. However, for an individual in poor condition, young birds, or during extreme weather events (e.g., very low temperatures), it is conceivable that the energy used to warm up in response to a “supermoon” event could have energetic implications for an individual.

## CONFLICT OF INTEREST

None declared.

## AUTHOR CONTRIBUTIONS

Conceptualization, S.J.P., P.B.F., and P.J.B.; methodology, S.J.P., C.R.W., P.B.F., J.A.G., and P.J.B; data collection, S.J.P. (cloud data) and P.J.B (all field and logger data); preliminary analysis, P.B.F.; formal analysis, C.R.W; writing, reviewing, and editing, S.J.P. wrote the first draft of the manuscript with input from all authors.

## ETHICAL APPROVAL

Work in Svalbard was undertaken with full permissions from the Governor of Svalbard and adhered to the Norwegian Animal Welfare Act. Although the United Kingdom Animal (Scientific Procedures) Act 1986 does not apply to Svalbard where this study was conducted, we were meticulous in following its provisions, especially those set out by the Home Office in the Official Guidance on the operation of the Act.

## Data Availability

All data are available as electronic supplementary material.
